# Supplementary material for: Towards unified AI-driven fracture mechanics: the extended deep energy method (XDEM)
Source: Nat Commun. 2026 Aug 15;17:8492. doi: 10.1038/s41467-026-76748-1 (PMC13484471; doi:10.1038/s41467-026-76748-1)
Supplement: Supplementary file 3 [file 41467_2026_76748_MOESM3_ESM.zip › pic/Results/Stress_intensity_factor/Mode2/crack2_differ_loads_contourf.pdf]

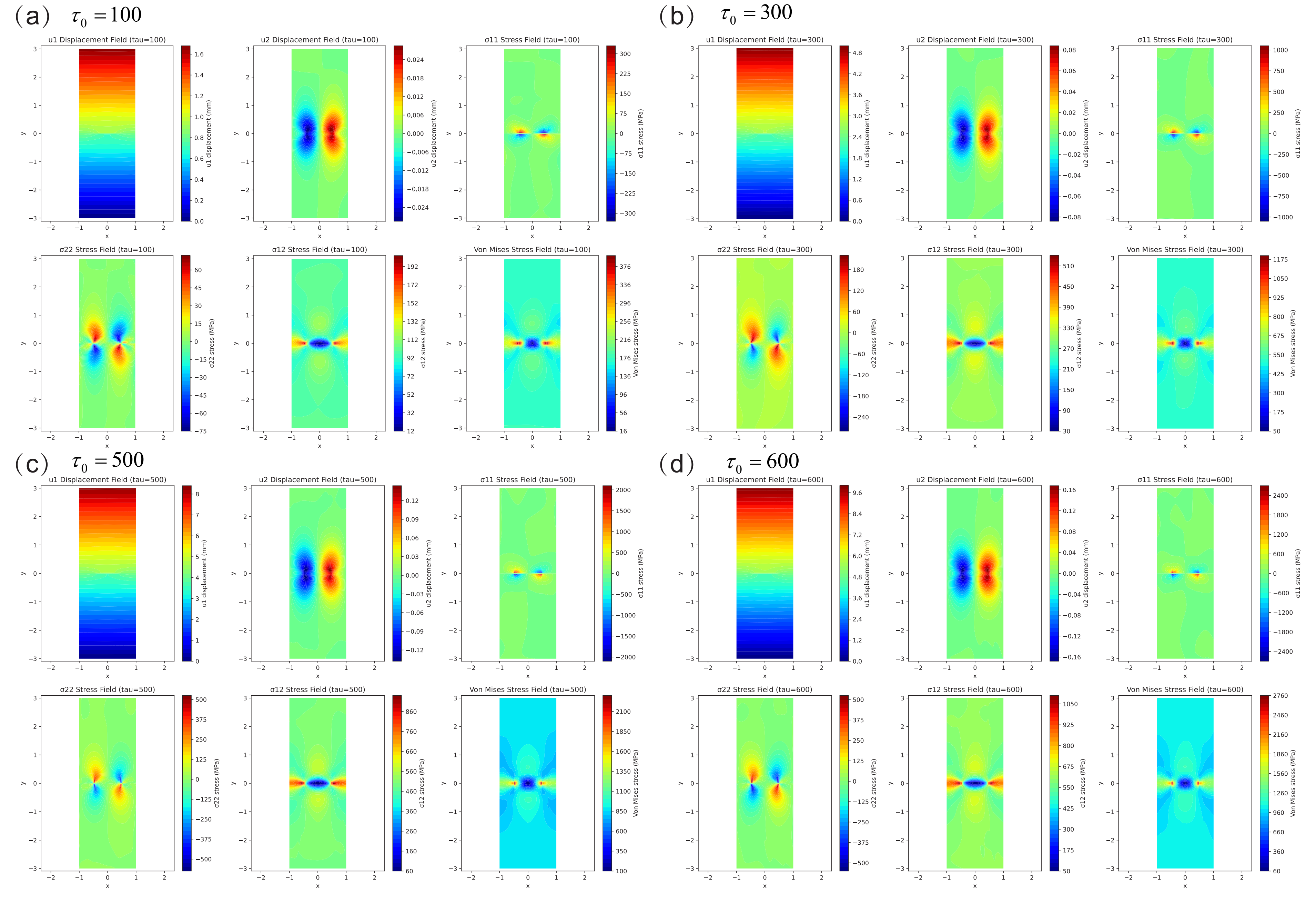

(a) $\tau_0 = 100$
u1 Displacement Field (tau=100)
u2 Displacement Field (tau=100)
σ11 Stress Field (tau=100)
σ22 Stress Field (tau=100)
σ12 Stress Field (tau=100)
Von Mises Stress Field (tau=100)
(b) $\tau_0 = 300$
u1 Displacement Field (tau=300)
u2 Displacement Field (tau=300)
σ11 Stress Field (tau=300)
σ22 Stress Field (tau=300)
σ12 Stress Field (tau=300)
Von Mises Stress Field (tau=300)
(c) $\tau_0 = 500$
u1 Displacement Field (tau=500)
u2 Displacement Field (tau=500)
σ11 Stress Field (tau=500)
σ22 Stress Field (tau=500)
σ12 Stress Field (tau=500)
Von Mises Stress Field (tau=500)
(d) $\tau_0 = 600$
u1 Displacement Field (tau=600)
u2 Displacement Field (tau=600)
σ11 Stress Field (tau=600)
σ22 Stress Field (tau=600)
σ12 Stress Field (tau=600)
Von Mises Stress Field (tau=600)
u1 displacement (mm)
u2 displacement (mm)
σ11 stress (MPa)
σ22 stress (MPa)
σ12 stress (MPa)
Von Mises stress (MPa)
x
y
